# A Novel Mechanical Metamaterial Exhibiting Auxetic Behavior and Negative Compressibility

**DOI:** 10.3390/ma13010079

**Published:** 2019-12-22

**Authors:** James N. Grima-Cornish, Joseph N. Grima, Daphne Attard

**Affiliations:** 1 Metamaterials Unit, Faculty of Science, University of Malta, Msida MSD 2080, Malta; joseph.grima@um.edu.mt; 2 Department of Chemistry, Faculty of Science, University of Malta, Msida MSD 2080, Malta

**Keywords:** auxetic, negative Poisson’s ratio, negative compressibility

## Abstract

Auxetics (negative Poisson’s ratio) and materials with negative linear compressibility (NLC) exhibit the anomalous mechanical properties of getting wider rather than thinner when stretched and expanding in at least one direction under hydrostatic pressure, respectively. A novel mechanism—termed the ‘triangular elongation mechanism’—leading to such anomalous behavior is presented and discussed through an analytical model. Amongst other things, it is shown that this novel mechanism, when combined with the well-known ‘rotating squares’ model, can generate giant negative Poisson’s ratios when the system is stretched.

## 1. Introduction

Most conventional materials share a number of macroscopic properties, such as the property of thinning when stretched (positive Poisson’s ratio), shrinking when placed under pressure (positive compressibility), or expanding when heated (positive thermal expansion coefficients) [1]. Nevertheless, not all materials behave in this way; in recent decades, there has been an increased focus on unconventional ‘negative’ materials [2,3,4,5,6,7,8,9,10,11,12,13,14,15,16,17,18,19,20,21,22,23,24,25,26,27,28,29,30,31,32,33,34,35,36,37,38,39,40,41,42,43,44,45,46,47,48,49,50,51,52,53,54,55,56,57,58,59,60,61,62,63,64,65,66,67,68,69,70,71,72,73,74,75,76,77]. These materials behave in the opposite manner. For example, auxetics exhibit the property of getting wider rather than thinner when uniaxially stretched [2]—a feature which corresponds to a negative Poisson’s ratio (NPR)—whilst materials with negative linear compressibility (NLC) expand in at least one direction when subjected to a compressive hydrostatic pressure [3]. Such behavior contrasts sharply with that manifested by most everyday materials, such as by common plastics or rubber, and has been associated with a number of enhanced mechanical properties, which make them preferable over their conventional counterparts in a number of practical applications which require the use of materials with superior properties [3,4,5,6,7,8,9,10]. For example, auxetics are known to perform better in applications ranging from cushioning (by naturally forming dome-shaped surfaces and offering enhanced resistance [4]) to noise and vibration control applications [5].

It is due to these enhanced properties and the wide range of applications that in recent years, there have been various studies which have attempted to design, model, synthesize, manufacture, or characterize materials and metamaterials which exhibit one or more of these anomalous properties. These include zeolites [11,12,13,14], silicates and other minerals [15], and polymeric systems [2,16,17,18,19,20,21,22] which exhibit negative Poisson’s ratios, in addition to metal organic frameworks (MOF) which exhibit negative linear compressibility [23,24,25]. In tandem, there has also been a significant amount of research aimed at exploring new mechanisms leading to anomalous behaviors. Examples of such mechanisms include the rotating rigid unit mechanisms [26,27,28,29,30,31,32], the umbrella-style mechanism [33,34,35], the re-entrant honeycomb deforming through hinging or flexure [35,36,37], etc. leading to NPR, as well as the wine-rack mechanism leading to NLC [23,24,25,38], together with the formulation of mathematical models to fully elucidate their behaviors [8,22,26,27,28,29,30,31,38].

Since it was first reported two decades ago [12], the rotating squares mechanism has established itself as one of the leading models which can explain auxeticity in a number of materials, where it has stood the test of time as a robust and highly applicable model—e.g., [14,21,32]. Variations of it have been produced [27,28,29,30,40,41], some of which have also served as models to explain why a number of naturally occurring materials (e.g., α-cristobalite and natrolite [14,42]) exhibit negative Poisson’s ratios. Nevertheless, this model has the limitation of being a two-dimensional (2D) model which—despite its strengths in explaining the behaviors of materials in particular projections—needs to be extended to generate negative Poisson’s ratios in all three dimensions (3D). The present work aims to address this lacuna by proposing and modeling a novel 3D system (mechanical metamaterial) which uses the 2D ‘rotating squares’ model and expands it to a fully auxetic 3D system through the use of the newly proposed ‘triangular elongation mechanism’ (TEM), which can cause an increase in out-of-plane projection when the ‘rotating squares’ model is stretched open. It is also shown that, as proposed, this system can exhibit other anomalous negative properties, such as NLC. The objective of this work is to put forward and rigorously analyze a new design concept for the manufacture of ‘negative mechanical systems’ which incorporate an equally novel auxeticity-generating mechanism, the ‘TEM’, thus augmenting the range of known mechanisms for producing such anomalous behavior.

## 2. Materials and Methods

### 2.1. The System Studied

**Figure 1:**
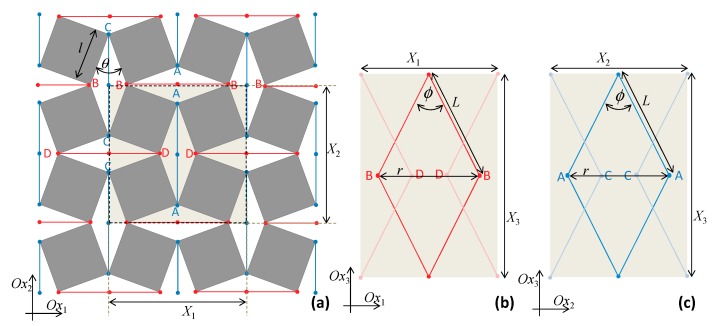
The system being modeled where (**a**) shows the *Ox*_1_–*Ox*_2_ projections with the unit cell highlighted whilst (**b**,**c**) show the *Ox*_1_–*Ox*_3_ and *Ox*_2_–*Ox*_3_ projections of the unit cell.

**Figure 2:**
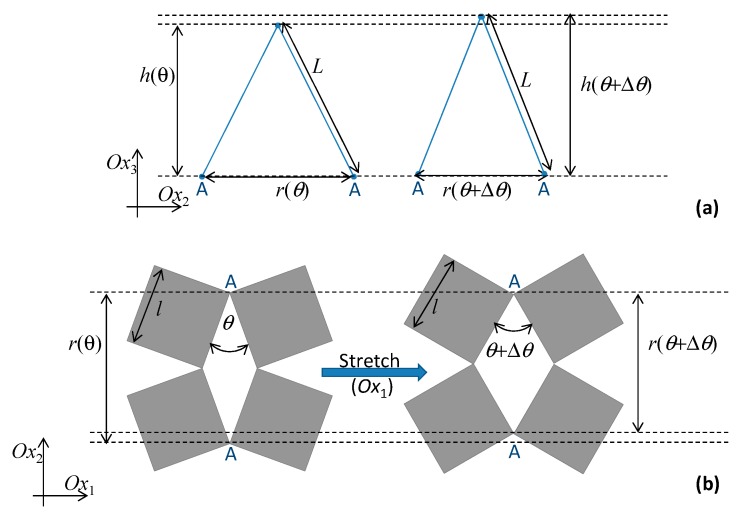
The auxetic-generating ‘triangular elongation mechanism’ being proposed; illustrated in (**a**) through a triangular building block where the base corresponds to the distance between two opposite vertices in the pore of the ‘rotating squares’ structure shown in (**b**). Note that as the system is stretched in the *Ox*_1_ direction, the square units located in the *Ox*_1_–*Ox*_2_ plane rotate relative to each other from an angle *θ* to *θ* + d*θ* with the consequence that the distance between opposite vertices A and A (=*r*) decreases. This forces the triangular units located in the orthogonal plane to elongate, causing an increase in *h* and generating a negative Poisson’s ratio.

The system studied in this work is represented in Figure 1 and may be described as an enhanced three-dimensional version of the well-known two-dimensional ‘rotating squares’ model. The enhancement is produced through extra elements which cause increases in out-of-plane dimensions as the system is stretched, thus converting it to a three-dimensional auxetic. These extra elements can be thought of as two sides of a triangular unit lying perpendicularly to the rotating squares structure (the base), attached at points that are brought closer together when the ‘rotating squares’ are stretched open. From a geometric perspective, the mechanism presented here is a consequence of the fact that, in triangular units, the height of a triangle must increase if the base length decreases, thus generating added thickness out-of-plane (a negative out-of-plane Poisson’s ratio due to what may be referred to as the ‘triangular elongation mechanism’) to accompany the well-known –1 Poisson’s ratio in-plane. This results in a metamaterial which exhibits a negative Poisson’s ratio both in-plane and out-of-plane. Due to practical considerations, the base attachment points shall be taken as the locations of two opposite vertices of the rhombic pores, i.e., where the hinges are located, marked as AA in pore ADAD, BB in pore ABAB, CC in pore BCBC, and DD in pore CDCD (see Figure 1). This means that the bases of the triangular units would correspond to the diagonals of the pores which are opposite pore angles π − *θ*, which—as clearly illustrated in Figure 2—get shorter whenever *θ* increases within the range of 0 to π/2, thus causing the required increase in height of the out-of-plane triangular units. The inspiration to use ‘TEM’ to achieve the out-of-plane deformation came from the ability of a triangular unit to transform from a perfectly flat structure to a fully erect one with a very significant and measurable change in height. All of this is triggered simply through a reduction of the direct distance between the ends, AA or BB. Furthermore, as implemented in the construction (see Figure 1) triangular elements with a single degree of freedom are simple enough to model analytically. Moreover, the inspiration to use the ‘rotating squares’ as a base structure comes from the fact that, apart from having the required features which may cause the TEM to operate (referring to Figure 1, the distances AA and BB in this model decrease as the system is pulled open by uniaxial stretching), it benefits from in-plane isotropic behavior, robustness, and implementability, as discussed in more detail below.

Referring to Figure 1, the system being studied may be represented through a periodic orthorhombic unit cell where the rotating squares structure is placed in the *Ox*_1_–*Ox*_2_ plane. With this alignment, the projections *X_i_* of the unit cell in the *Ox_i_* directions are given by:

(1) $$X_{1}=X_{2}=2\sqrt{2}l\sin\left(\frac{\pi }{4}+\frac{\theta }{2}\right)$$

(2) $$X_{3}=2h$$

where *h* is the height of the out-of-plane triangular units, given by:

(3) $$h=\sqrt{L^{2}-{\left(\frac{r}{2}\right)}^{2}},$$

where *L* is the length of the additional elements forming the sides of the triangular units which move in the third dimension and *r* is the base length of the triangles which correspond to the aforementioned diagonals of the rhombic pores (opposite the angle π − *θ*) that, for *θ* between 0° and π/2, will be getting shorter as the rotating squares structure is pulled open (*θ* increases). Note that the factor of 2 is necessary, since it is being assumed that triangular units are located both above and below the plane of the ‘rotating squares’, where they could either have the same base—in which case the aforementioned triangular unit would be half of a rhombic structure, having diagonals of length *r* and 2*h* (see Figure 1)—or at different locations. The use of rhombic units, having triangular units both above and below the ‘rotating squares base’ and forming a wine-rack/Milton’s unimode-like system [31,43,44], has the advantage that the system is easily tessellatable and will not shear. Note also that, assuming that the squares are perfectly rigid (as is normally assumed in the idealized ‘rotating squares’ model), the length *r* is a function of the single variable *θ*, the angles between the squares, and is given by:

(4) $$r=2l\cos\left(\frac{\theta }{2}\right),$$

i.e., the height *h* of the triangular unit may be written as:

(5) $$h=\sqrt{L^{2}-l^{2}\cos^{2}\left(\frac{\theta }{2}\right)},$$

or, in terms of *λ*, the ratio of lengths *L*:*l*, defined as:

(6) $$\lambda =\frac{L}{l}.$$

We obtain:

(7) $$h=l\sqrt{\lambda^{2}-\cos^{2}\left(\frac{\theta }{2}\right)},$$

and projections *X*_3_ of the unit cell in the *Ox*_3_ direction are given by:

(8) $$X_{3}=2l\sqrt{\lambda^{2}-\cos^{2}\left(\frac{\theta }{2}\right)}.$$

Note that for physically realizable systems, for a triangle to form, it is required that:

(9) $$L>\frac{r}{2},$$

where the special case *L* = *r*/2 relates to the system adopting a hypothetical perfectly flat conformation which would correspond to an angle between the squares of $\theta_{f}$ and have zero out-of-plane thickness (i.e., *de facto* nonexistent). In practice, this means that *L* may approach *r*/2 but may not be equal to it. This special limiting angle of $\theta_{f}$ may be expressed in terms of the other geometric parameters (the ratio of the lengths of the ligaments to the side lengths of the squares) as follows:

(10) $$\theta_{f}=2\cos^{-1}\left(\lambda \right).$$

This flat conformation will only exist if *L* < *l*, with *L = l* corresponding to a very special—once again hypothetical—case where the system is fully flat when *θ* *= 0* (i.e., when the rotating squares structure is fully closed). Note also that, unless restricted by the length of *L* (see Equation (7)), whilst *θ* can physically attain a maximum range of 0 ≤ *θ* ≤ π, for the purpose of this work, unless otherwise stated, it shall be assumed that 0 ≤ *θ* < π/2 so as to ensure that in the *Ox*_1_–*Ox*_2_ plane, stretching will cause an increase in the value of *θ* and a decrease in the value of *r*, as required for the auxetic ‘triangular elongation mechanism’ to operate. Furthermore, in cases when $\theta_{f}\neq 0$, if it is assumed that the elements of length *L* are not stretchable, then the conformation when $\theta =\theta_{f}\neq 0$ (a hypothetical system as this would have a zero thickness, i.e., does not exist) is the most closed conformation that the system can attain, since this would effectively result in a physically locked conformation with the added ligaments acting as ‘brakes’ for the rotational movements. This means that for the ‘triangular elongation mechanism’ leading to out-of-plane auxetic behavior to operate, the system is physically constrained to existing with the following range of angles between the squares:

(11) $$\theta_{f}<\theta <\pi /2\Rightarrow 2\cos^{-1}\left(\lambda \right)<\theta <\pi /2$$

where the special case when *λ* = 1 (i.e., *L = l*) gives the maximum range for auxeticity from 0 to π/2. Values of θ>π/2 correspond to conventional out-of-plane behavior. 

### 2.2. Analytical Model

With all of this in mind, it is possible to derive expressions for the Poisson’s ratios of the system, which are the negatives of the ratios of lateral strain to axial strain. In the scientific literature, there is more than one way to formally define the Poisson’s ratio, which essentially depends on the manner how strain is measured. All of the forms that are in use have their particular advantages over others in particular circumstances. One of the more commonly used formulations of the Poisson’s ratio is to define it at particular conformations (i.e., in this case, as a function of *θ*) at infinitesimally small strains. This form of the Poisson’s ratio is sometimes referred to as the Poisson’s function or the instantaneous Poisson’s ratio, and has the advantage that it gives a measure of what the Poisson’s ratio is at the exact instant of observation as the system is deforming. This may be particularly useful when a system would be changing the manner in which it is deforming, as is the case of the hinging honeycomb when transforming to the non-reentrant form from the reentrant one upon stretching. The form of the Poisson’s ratio used would ideally be able to capture that the system would no longer be getting wider once it goes past the reentrant to non-reentrant transition point. Other formulations include, for example, the engineering Poisson’s ratio, defined as the negative of the ratio of the engineering strains, which is very simple to compute and is particularly appealing for applications where the initial and final states of the material are of relevance, as is normally the case in real practical applications.

From a computational perspective, since the system can be defined in terms of the single variable *θ* in this case, the Poisson’s function may be computed from the ‘infinitesimally small strains’ *dε_i_* in the *Ox_i_* directions when changing the angle from *θ* to *θ* + *dθ*; it may be obtained by differentiating expressions (1) and (8) with respect to *θ* so as to obtain the strains as follows:

(12) $$d\varepsilon_{i}=\frac{1}{X_{i}}\left(\frac{dX_{i}}{d\theta }\right)d\theta .$$

The expressions for the Poisson’s ratio *ν_ij_* in the *Ox_i_–Ox_j_* plane for stretching in the *Ox_i_* direction, theoretically valid for *θ*_f_ < *θ* < π/2 and π/2 < *θ* < π (see discussion below)—in analogy to the work of Grima and Evans (2000) [26]—in the limit of zero strain for a given value of *θ* may be obtained as follows:

(13) $$\nu_{ij}=-\frac{d\varepsilon_{j}}{d\varepsilon_{i}}=-\frac{X_{i}}{X_{j}}\left(\frac{dX_{j}}{d\theta }\right){\left(\frac{dX_{i}}{d\theta }\right)}^{-1}.$$

In analogy to the work of Prall and Lakes [45], Grima and Evans [26], and others, the moduli of this system may be computed using an energy conservation approach in terms of the stiffness associated with the hinges, where one may consider one of three ways to how the stiffness is introduced:

- The stiffness is the result of changes in the angles *θ* between the squares with the out-of-plane elements being connected through pin-joints which offer no resistance.
- The stiffness is the result of changes in the out-of-plane angles *ϕ* between the ligaments with the squares being connected though frictionless hinges.
- Both the in-plane and out-of-plane hinges associated with the angles *θ* and *ϕ* offer resistance.

In the latter, more general case, since the unit cell as defined in Figure 1 contains eight *θ* and eight *ϕ* ‘spring hinges’, the total work done per unit cell when *θ* becomes *θ* + *dθ* and *ϕ* becomes *ϕ + dϕ* may be expressed as:

(14) $$W=8\left[\frac{1}{2}k_{\theta }{\left(d\theta \right)}^{2}\right]+8\left[\frac{1}{2}k_{\phi }{\left(d\phi \right)}^{2}\right]$$

where, recognizing that *θ* and *ϕ* are interdependent variables, *dθ* and *dϕ* are interrelated, i.e., *dϕ* may be expressed in terms of *dθ*. In fact, recognizing that the parameters *r* and *h* defined in Equations (4) and (5) may also be written in terms of *ϕ* as:

(15) $$r=2L\sin\left(\frac{\phi }{2}\right)$$

and:

(16) $$h=L\cos\left(\frac{\phi }{2}\right),$$

then *dθ* and *dϕ* on *dr*, the change in *r*, must be recognized through:

(17) $$dr=-l\sin\left(\frac{\theta }{2}\right)d\theta =L\cos\left(\frac{\phi }{2}\right)d\phi =hd\phi ,$$

i.e.,:

(18) $$d\phi =-\frac{l\sin\left(\frac{\theta }{2}\right)}{\sqrt{L^{2}-l^{2}\cos^{2}\left(\frac{\theta }{2}\right)}}d\theta =-\frac{\sin\left(\frac{\theta }{2}\right)}{\sqrt{\lambda^{2}-\cos^{2}\left(\frac{\theta }{2}\right)}}d\theta .$$

In the special case when *λ* = 1 (*L* = *l*), this simplifies to:

(19) dϕ=−dθ.

which could have been obtained directly by recognizing that, in such a case:

(20) $$r=2l\cos\left(\frac{\theta }{2}\right)=2l\sin\left(\frac{\phi }{2}\right)=2l\cos\left(\frac{\pi }{2}-\frac{\phi }{2}\right)\Rightarrow \theta =\pi -\phi .$$

Thus, in the general case, the work done per unit cell can be re-expressed in terms of dθ as follows:

(21) $$\begin{array}{ll} W & =8\left[\frac{1}{2}k_{\theta }{\left(d\theta \right)}^{2}\right]+8\left[\frac{1}{2}k_{\phi }{\left(\frac{-\sin\left(\frac{\theta }{2}\right)}{\sqrt{\lambda^{2}-\cos^{2}\left(\frac{\theta }{2}\right)}}d\theta \right)}^{2}\right]\\ & =\left[4k_{\theta }+4k_{\phi }\frac{\sin^{2}\left(\frac{\theta }{2}\right)}{\lambda^{2}-\cos^{2}\left(\frac{\theta }{2}\right)}\right]{\left(d\theta \right)}^{2} \end{array}$$

In the specific case where *L* = *l:*

(22) $$\begin{array}{ll} W & =8\left[\frac{1}{2}k_{\theta }{\left(d\theta \right)}^{2}\right]+8\left[\frac{1}{2}k_{\phi }{\left(-d\theta \right)}^{2}\right]\\ & =4\left[k_{\theta }+k_{\phi }\right]{\left(d\theta \right)}^{2} \end{array}$$

We may also write an expression for *U*, the strain energy per unit volume, in terms of is *dθ* as follows:

(23) $$U=\frac{1}{2}E_{i}{\left(d\varepsilon_{i}\right)}^{2}=\frac{1}{2}E_{i}{\left[\frac{dX_{i}}{X_{i}}\right]}^{2}=\frac{1}{2}E_{i}{\left[\frac{1}{X_{i}}\left(\frac{dX_{i}}{d\theta }\right)d\theta \right]}^{2},$$

which can be related to *W*, through the principle of conservation of energy:

(24) $$U=\frac{1}{V}W,$$

where *V* is the volume of the unit cell given by:

(25) $$V=X_{1}X_{2}X_{3}.$$

Thus, from the equations above, we obtain, for the general case:

(26) $$\frac{1}{2}E_{i}{\left[\frac{1}{X_{i}}\left(\frac{dX_{i}}{d\theta }\right)d\theta \right]}^{2}=\frac{1}{X_{1}X_{2}X_{3}}\left[4k_{\theta }+4k_{\phi }\frac{\sin^{2}\left(\frac{\theta }{2}\right)}{\lambda^{2}-\cos^{2}\left(\frac{\theta }{2}\right)}\right]{\left(d\theta \right)}^{2},$$

which, making *E_i_* the subject of the formula and simplifying, gives an expression for *E_i_* as follows:

(27) $$E_{i}=\frac{8X_{i}^{2}}{X_{1}X_{2}X_{3}}\left[k_{\theta }+k_{\phi }\frac{\sin^{2}\left(\frac{\theta }{2}\right)}{\lambda^{2}-\cos^{2}\left(\frac{\theta }{2}\right)}\right]{\left(\frac{dX_{i}}{d\theta }\right)}^{-2}.$$

In the special case when *L* = *l*, this simplifies to:

(28) $$E_{i}=\frac{8X_{i}^{2}}{X_{1}X_{2}X_{3}}\left[k_{\theta }+k_{\phi }\right]{\left(\frac{dX_{i}}{d\theta }\right)}^{-2}.$$

#### 2.2.1. The Poisson’s Ratios

From Equations (1), (4), and (8), it may be shown that the Poisson’s ratios may be simplified as follows:

(29) $$\nu_{12}={\left(\nu_{21}\right)}^{-1}-\frac{d\varepsilon_{2}}{d\varepsilon_{1}}=-\frac{\cot\left(\frac{\pi }{4}+\frac{\theta }{2}\right)}{\cot\left(\frac{\pi }{4}+\frac{\theta }{2}\right)}=\{\begin{array}{cc} -1 & 0<\theta <\pi ,\theta \neq \frac{\pi }{2}\\ undefined & \theta =\frac{\pi }{2} \end{array}$$

and:

(30) $$\nu_{13}=\nu_{23}={\left(\nu_{31}\right)}^{-1}={\left(\nu_{32}\right)}^{-1}=-\frac{d\varepsilon_{3}}{d\varepsilon_{1}}=-\frac{d\varepsilon_{3}}{d\varepsilon_{2}}=-\frac{\sin\left(\theta \right)\tan\left(\frac{\pi }{4}+\frac{\theta }{2}\right)}{2\left(\lambda^{2}-\cos^{2}\left(\frac{\theta }{2}\right)\right)}.$$

#### 2.2.2. The Young’s Moduli

From Equations (1), (4) and (19), it may be shown that the Young’s moduli may be simplified as follows:

(31) $$E_{i}=\frac{8}{X_{3}}\left[k_{\theta }+k_{\phi }\frac{\sin^{2}\left(\frac{\theta }{2}\right)}{\lambda^{2}-\cos^{2}\left(\frac{\theta }{2}\right)}\right]{\left(\frac{dX_{i}}{d\theta }\right)}^{-2}(i=1,2)$$

and

(32) $$E_{3}=\frac{8X_{3}^{}}{X_{1}X_{2}}\left[k_{\theta }+k_{\phi }\frac{\sin^{2}\left(\frac{\theta }{2}\right)}{\lambda^{2}-\cos^{2}\left(\frac{\theta }{2}\right)}\right]{\left(\frac{dX_{3}}{d\theta }\right)}^{-2},$$

i.e.,:

(33) $$E_{1}=E_{2}=2\left[k_{\theta }+k_{\phi }\frac{\sin^{2}\left(\frac{\theta }{2}\right)}{\lambda^{2}-\cos^{2}\left(\frac{\theta }{2}\right)}\right]\;{\left[l^{3}\cos^{2}\left(\frac{\pi }{4}+\frac{\theta }{2}\right)\left(\sqrt{\lambda^{2}-\cos^{2}\left(\frac{\theta }{2}\right)}\right)\right]}^{-1}$$

and:

(34) $$E_{3}=8\left[k_{\theta }+k_{\phi }\frac{\sin^{2}\left(\frac{\theta }{2}\right)}{\lambda^{2}-\cos^{2}\left(\frac{\theta }{2}\right)}\right]\;\frac{{\left(\lambda^{2}-\cos^{2}\left(\frac{\theta }{2}\right)\right)}^{3/2}}{l^{3}\sin^{2}\left(\theta \right)\sin^{2}\left(\frac{\pi }{4}+\frac{\theta }{2}\right)}.$$

In the special case when *λ* = 1 (i.e., *L = l*):

(35) $$E_{1}=E_{2}=2\left[k_{\theta }+k_{\phi }\right]\;{\left[l^{3}\cos^{2}\left(\frac{\pi }{4}+\frac{\theta }{2}\right)\sin\left(\frac{\theta }{2}\right)\right]}^{-1}(i=1,2)$$

and:

(36) $$E_{3}=8\left[k_{\theta }+k_{\phi }\right]\;\left[\frac{\sin^{3}\left(\frac{\theta }{2}\right)}{l^{3}\sin^{2}\left(\theta \right)\sin^{2}\left(\frac{\pi }{4}+\frac{\theta }{2}\right)}\right].$$

Note that equations *E*_1_ and *E*_2_ in Equation (33) will tend to infinity as θ tends to π/2, the fully open position, as expected, since at this instance, the system becomes locked. This is not the case for *E*_3_, thus indicating that one may go past the π/2 locking position through a process of stretching or compressing in the *Ox*_3_ direction.

#### 2.2.3. The Compliance Matrix, Linear Compressibility, and Off-Axis Properties

Recognizing that, assuming idealized hinging behavior, the system as designed cannot shear on-axis, it may be further deduced that the only non-zero elements of the 6 × 6 compliance **S** = (*s_ij_*) (*i*,*j* = 1,2,…6) matrix are *s_ij_* (*i*,*j* = 1,2,3). Thus, the compliance matrix of this system is given by:

(37) $$S=\left(\begin{array}{cccccc} s_{11} & s_{12} & s_{13} & 0 & 0 & 0\\ s_{21} & s_{22} & s_{23} & 0 & 0 & 0\\ s_{31} & s_{32} & s_{33} & 0 & 0 & 0\\ 0 & 0 & 0 & 0 & 0 & 0\\ 0 & 0 & 0 & 0 & 0 & 0\\ 0 & 0 & 0 & 0 & 0 & 0 \end{array}\right)=\left(\begin{array}{cccccc} \frac{1}{E_{1}} & \frac{-\nu_{21}}{E_{2}} & \frac{-\nu_{31}}{E_{3}} & 0 & 0 & 0\\ \frac{-\nu_{12}}{E_{1}} & \frac{1}{E_{2}} & \frac{-\nu_{32}}{E_{3}} & 0 & 0 & 0\\ \frac{-\nu_{13}}{E_{1}} & \frac{-\nu_{23}}{E_{2}} & \frac{1}{E_{3}} & 0 & 0 & 0\\ 0 & 0 & 0 & 0 & 0 & 0\\ 0 & 0 & 0 & 0 & 0 & 0\\ 0 & 0 & 0 & 0 & 0 & 0 \end{array}\right).$$

The elements of this matrix may be used to obtain the on-axis compressibility, which defines how the system responds to a hydrostatic pressure *p*, which, in the *Ox_i_* direction, is defined by:

(38) $$\beta_{i}=s_{i1}+s_{i2}+s_{i3}i=1,2,3,$$

as well as the off-axis mechanical properties, which may be obtained using standard axis transformation techniques as discussed elsewhere [78].

## 3. Results and Discussion

**Figure 3:**
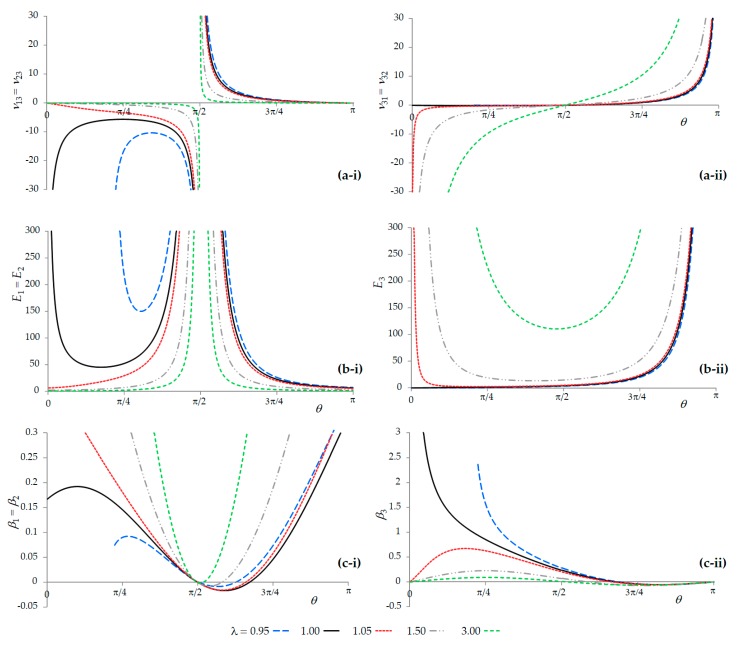
Plots of (**a**) the Poisson’s ratio in the *Ox*_1_–*Ox*_3_ and *Ox*_2_–*Ox*_3_ planes for loading in (i) the *Ox*_1_ or *Ox*_2_ direction and in (ii) the *Ox*_3_ direction; (**b**) the Young’s moduli in (i) the *Ox*_1_ or *Ox*_2_ direction and in (ii) the *Ox*_3_ direction; and (**c**) the linear compressibility in (i) the *Ox*_1_ or *Ox*_2_ direction and in (ii) the *Ox*_3_ direction. Note that negative Poisson’s ratios in the *Ox*_1_–*Ox*_3_ and *Ox*_2_–*Ox*_3_ planes are always present when *θ* is less than π/2, whilst negative compressibility is present when *θ* is greater than π/2. Note that for these plots, *k_ϕ_* and *k_ϕ_* were arbitrarily chosen as ½ and 1, respectively, whilst *l* was set as 1. Units are arbitrary.

Plots of the Poisson’s ratios, Young’s moduli, and compressibility (on-axis) for the various values of *λ* are shown in Figure 3. These plots clearly show that the system, as modified through the addition of the extra out-of-plane ligaments, exhibits various properties which could not be afforded by its two-dimensional counterpart, such as the six simultaneously negative Poisson’s ratios *ν_ij_* (*i*,*j* = 1,2,3) which are exhibited when *θ* is less than π/2, the possibility of passing through the ‘locking conformation’ through stretching/compression in the *Ox*_3_ direction, and the negative linear compressibility in some systems when *θ* is greater than π/2. In fact, using the nomenclature coined by Wojciechowski, this system may be termed as a ‘complete auxetic’, meaning that it may exhibit a negative Poisson’s ratio for loading in any direction [69,70] (as opposed to partially auxetic, which refers to systems which are only auxetic in specific planes for loading in specific directions, or non-auxetic, which refers to systems which do not exhibit any auxetic behavior). Certain other important features, such as the −1 Poisson’s ratios for in-plane loading in the *Ox*_1_–*Ox*_2_ plane, are retained. While the plots of the various properties as illustrated in Figure 3 are self-explanatory and highlight the versatility of the system as a truly multifunctional metamaterial which can exhibit a number of anomalous negative properties, it would be useful to discuss some of the other aspects that emerge from the expressions.

A particular feature of this model which merits discussion includes the stiffness characteristics and how they compare with those of the original 2D analogue by Grima and Evans (2000) [26]. For example, it should be noted that the moduli *E*_1_ and *E*_2_ of the system presented here, had the stiffness be imparted only through the in-plane ‘rotating squares’ *θ*-hinges, i.e., *k_ϕ_* = 0, are not equivalent to those derived for the original 2D analogue. This is due to the fact that, upon stretching, the stiffness of the model is being attenuated by the very large increase in the dimensions in the *Ox*_3_ dimension and vice-versa during compression. Obviously, to make a fair comparison, it is important that the monolayer 2D system is converted to a multilayer system (as in the relationship between graphene and graphite) and that *L* is chosen in a manner that gives the same density of squares for the initial conformation. In addition, the tendency to get the moduli tending to +∞ as the present system approaches its flat conformation (zero third dimensional thickness) is a property which could not be achieved by systems having a constant out-of-plane thickness.

The very pronounced change in separations between the parallel sets of squares tessellated down the *Ox*_3_ dimension, which may be induced through a rotation of the squares or pulling of the structure, may also lend itself to a number of practical applications, which may range from mechanically tunable electronic components to smart erectable structures. It suffices to mention the fact that the systems where *λ* < 1 are expected to be erected from a perfectly flat conformation to one with a very significant thickness, where the systems with *λ*→1 could change their unit cell dimensions from circa 2l×2l×0 (a flat sheet, when *θ* = 0) to 2√2l×2√2l×√2l (a cuboid, when *θ* = π/2) to 2l×2l×2l (a cube, when *θ* = π/2) as *θ* changes from 0 to π. Obviously, the reverse could also happen, as it is possible to compress from a cube to a flat sheet.

Another feature that is noteworthy is the fine manner of how the stiffness in the structure can be controlled. In fact, although the *dθ* and *dϕ* are not independent of each other, the relationship between them is a rather complex one which depends on both the value of *θ* (or *ϕ*)—i.e., the extent of the aperture of the system—and the ratio *λ*. This means that through the careful choice of the geometric parameters used and the relative extents of *k_θ_* and *k_ϕ_*, one may fine-tune the stiffness of the system, as well as the particular shape of the stiffness vs. *θ* relationship (which dictates the shape of the stress–strain plot) for particular practical applications.

Focusing on the Poisson’s ratio, it is important to emphasize that the regions of the positive out-of-plane Poisson’s ratio when *θ* is greater than π/2 should not be viewed as a limitation, as it is this property which results in the negative compressibility behavior, a feature which is shared with other anisotropic NLC materials where a high positive Poisson’s ratio results in NLC. In addition, the physical co-existence of the ‘rotating squares’ and ‘wine-rack’/‘unimode’ mechanisms, which are well known for other negative thermo-mechanical properties such as negative thermal expansion (NTE) [46,47,48,49,50], permits the potential co-existence of multiple anomalous thermo-mechanical properties which are beyond the scope of this work to investigate exhaustively. 

**Figure 4:**
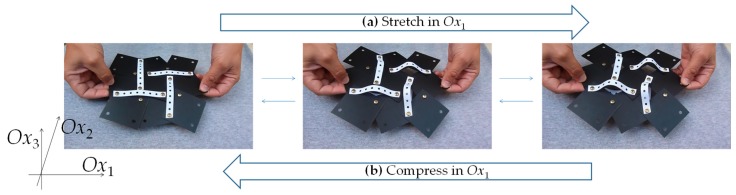
Image showing a ‘monolayer’ physical prototype (**a**) being stretched in the *Ox*_1_ direction or (**b**) compressed. Note that on stretching in *Ox*_1_, there is an increase in width in the *Ox*_2_ direction due to the rotating squares mechanism and an increase in thickness in the *Ox*_3_ direction due to the proposed triangular elongation mechanism (TEM), which is implemented here through thin flat ligaments which flex out of plane (see the animation). Note also that, despite the crudeness in construction of the physical prototype (squares made from regular sheets of *c*. 0.4 mm plastic, TEM implanted via a thin sheet of perforated paper *c*. 0.1 mm in thickness and 90 mm in length, and connections provided by regular office brass fasteners), there is still a remarkable similarity with the idealized hinging model presented above.

Before we conclude, it is important to highlight some important aspects which should be considered if the concepts presented here are to be used for the design and manufacture of physical materials or mechanical materials in real practical applications. First and foremost, it is important to highlight that although the present work has relied on the use of the rotating squares system as the ‘base structure’ of this novel metamaterial, other systems which have within them the essential minimal structural features that can support the proposed ‘triangular elongation mechanism’ (TEM) can also be used. This means that systems such as those of the rotating rectangles, parallelograms, rhombi, or triangles, as well as the classical wine-rack model and a number of honeycombs, can all potentially be transformed into three-dimensional auxetic metamaterials of this form. In addition, although this work has relied on hinging via a unimode wine-rack style mechanism for the out-of-plane deformation, one could conceive other ‘designs’ that can be ‘pushed out of plane’. For example, as shown in Figure 4, one can achieve the same effect through the use of thin flexible ligaments or sheets which are pushed from both ends, forcing them to buckle and deflect laterally (provided that the applied load exceeds the critical buckling load). Irrespective of the exact shape of the deflection curve (which is not the scope of this study), the extent of lateral deflection in such systems is generally not negligible and arises due to the fact that flexure/hinging modes of deformations are generally preferred to stretching/compression. It should also be noted that although the model presented here is in the form of a 3D fully periodic system, the same mechanism can also be implemented as a 2D sheet-like system (which could be considered as a model for a thin-sheet material) or as a finite system. All of these different forms of potential implementation make this material model even more versatile as a blueprint for the design of new materials and metamaterials. To realize the merits of the proposed structure and to have a direct proof of the versatility of the proposed system and its ability to achieve large negative Poisson’s ratios out-of-plane, we directly measured the deformations that occurred when the prototype shown in Figure 4 was stretched (see Animation in Supplementary Information). These measurements confirm a change in the out-of-plane dimensions from *c*. 1 mm to *c*. 40 mm as the system is pulled in the *Ox*_1_ direction from a characteristic length of *c*. 120 mm to *c*. 135 mm, a change which corresponds to an engineering Poisson’s ratio of *c*. −9.

Furthermore, as evident from the expressions derived, the properties reported here are not dependent on the actual scale of the system or on the chemical composition of the materials used for the construction, as is the case with other mechanical metamaterials. This important property means that the blueprint discussed here could, at least in theory, be fully implemented at any scale of structure, down to the nanoscale, where real materials could be designed in a manner that they mimic the behavior of the systems presented here. It is comforting to note that in recent years, there have already been a number of attempts to design, synthesize, and characterize a number of materials based on either the ‘rotating squares’ or the ‘wine-rack’/’unimode’ mechanisms. However, it should also be remembered that the model presented here is a highly idealized model where, for example, the squares and the ligaments are treated as perfectly rigid units which rotate/hinge relative to each other without bending, warping, or stretching. In real physical implementations, such idealized behavior may not be as easily achieved and other modes of deformation are likely to be present. Whether such ‘extra’ modes would be significant enough to drastically reduce the extent of anomalous behavior reported here (possibly even annihilating it) remains to be seen. In this respect, it should be appreciated that the mathematical model presented here, particularly the compliance matrix reported here in Equation (37), has its limitations and should be used with some caution. First and foremost, this compliance matrix is only fully applicable for idealized systems deforming solely and exclusively through hinging; it assumes highly idealized operations (such as the use of perfectly elastic hinges, complete lack of imperfections, etc.). In real physical implementations, such perfection is difficult to achieve; e.g., one would not be able to attain a zero *s*_44_, *s*_55_, and *s*_66_, as in practice, infinite resistance to shear cannot be attained (at the very least, there are the limitations imposed by the intrinsic mechanical properties of the constituent materials). Apart from this, the matrix in Equation (37) predicts the properties at small strains (i.e., it gives a measure of the various properties for a particular conformation of the model at a specific instant in the deformation profile); this is not the ideal formulation to describe the behavior of the system as a function of strain. In addition, although auxetics are known to exist at the nano, micro, and macro scales, it may be more of a challenge to implement the presented mechanism at the “smaller” scales, and more research is needed to convert this theoretical model to a working prototype at the smaller length scales. Here, particular emphasis must be placed on how the TEM could be joined with rotating squares. In this respect, it would also be useful if additional modeling and computations are carried out to investigate the stresses in the system, particularly for cases when the Poisson’s ratio tends to the more extreme values. It is, however, comforting that existing experimental work on the ‘rotating squares’ or the ‘wine-rack’/’unimode’ mechanisms has so far confirmed that these mechanisms are very robust and normally implementable, thus auguring well for the possible future developments of multifunctional materials and metamaterials based on the model presented here. In addition, the macroscale prototype shown in Figure 4, which includes TEM components made of paper, seems to perform well.

Obviously, the work presented here should be viewed within the framework of other work in this field aimed at achieving negative mechanical properties in 3D. Here it should be mentioned that, apart from the planar rotational structures (for example, rotating rigid squares, parallelograms, triangles, etc. [26,27,28,29,30,31,32]), 3D rotational structures constructed of cubes linked at certain edges or vertices, either directly or through ligaments [71,72,73], can also be employed. When such systems are stretched or compressed, the cubic units rotate and angles between the adjacent sides change, leading to spatial expansion or shrinkage that may be conducive of auxetic behavior. Other 3D auxetics were recently put forward by Almgren [35], Ma et al. [74,75], and Lim [76], who proposed systems which may be described in terms of the 3D equivalents of the reentrant honeycombs [36,37] or the double arrowhead honeycomb structure [77]. Some of these systems can also exhibit giant negative Poisson’s ratios: For example, Lim [78] has shown, through a similar geometrical analysis on the basis of rigid linkages with rotational joints, that auxeticity in his model is manifested in all three orthogonal planes, in which the auxeticity may even tend to infinity. While recognizing that it is beyond the scope of this work to report a detailed review or comparison of the different systems, which all involve some rotating elements, it is worthwhile to mention that the applicability or otherwise of a particular design over another is dependent on a number of factors, which may range from ease of manufacture to particular design features which may make one system preferable to another. For example, the present system offers the advantage that it is made of plates and rods (or beams) rather than cuboids, which means that it is likely to be lighter than its counterparts constructed from cuboidal solid units. In addition, the presence of plates which remain parallel to each other throughout the deformation process (which are not present in the other systems) may be beneficial in specific applications which may require them (e.g., in the manufacture of smart electronic devices).

## 4. Conclusions

This paper has presented a novel concept for transforming the two-dimensional rotating squares mechanism into a three-dimensional construct which can exhibit a multitude of anomalous negative properties. It was shown that one may make use of the feature that upon stretching of the ‘rotating squares’ base structure, the distance between some of the opposite hinged corners actually decreases so as to put into operation a newly proposed ‘triangular elongation mechanism’ (TEM) to generate negative out-of-plane Poisson’s ratios. With the help of analytical models, it was formally proven that this system is capable of demonstrating six simultaneously negative Poisson’s ratios *ν_ij_* (*i*,*j* = 1,2,3), as well as negative linear compressibilities, both of which are scale-independent properties and thus implementable at any scale of structure. Other ways of constructing these systems, such as the possible use of different base structures (e.g., rotating rectangles, triangles, parallelograms, rhombi, wine-racks, or honeycombs) in lieu of the ‘rotating squares’ were also discussed. Given the versatility of the model used—which is even applicable to sheet-like materials—together with the well-known fact that the mechanisms used here may also manifest other negative thermo-mechanical properties such as negative thermal expansion, we hope that this work will provide an impetus to experimentalists to design and manufacture new materials and metamaterials which mimic the model presented here or variations of it. We are also confident that some of the innovative contributions disclosed in this work—particularly the ‘TEM’ concept, the prototype itself, and the elucidation of how the TEM and the rotating rigid units mechanisms can be made to work together in a synergistic manner—has helped expand the state of the art in the field of mechanical metamaterials. 

## Supplementary Materials

The following are available online at https://www.mdpi.com/1996-1944/13/1/79/s1. Figure S1: Animation of the Proposed Auxetic Mechanism.
