# Supplementary figures and images for: A Novel Mechanical Metamaterial Exhibiting Auxetic Behavior and Negative Compressibility
Source: Materials (Basel). 2019 Dec 22;13(1):79. doi: 10.3390/ma13010079 (PMC6982174; doi:10.3390/ma13010079)

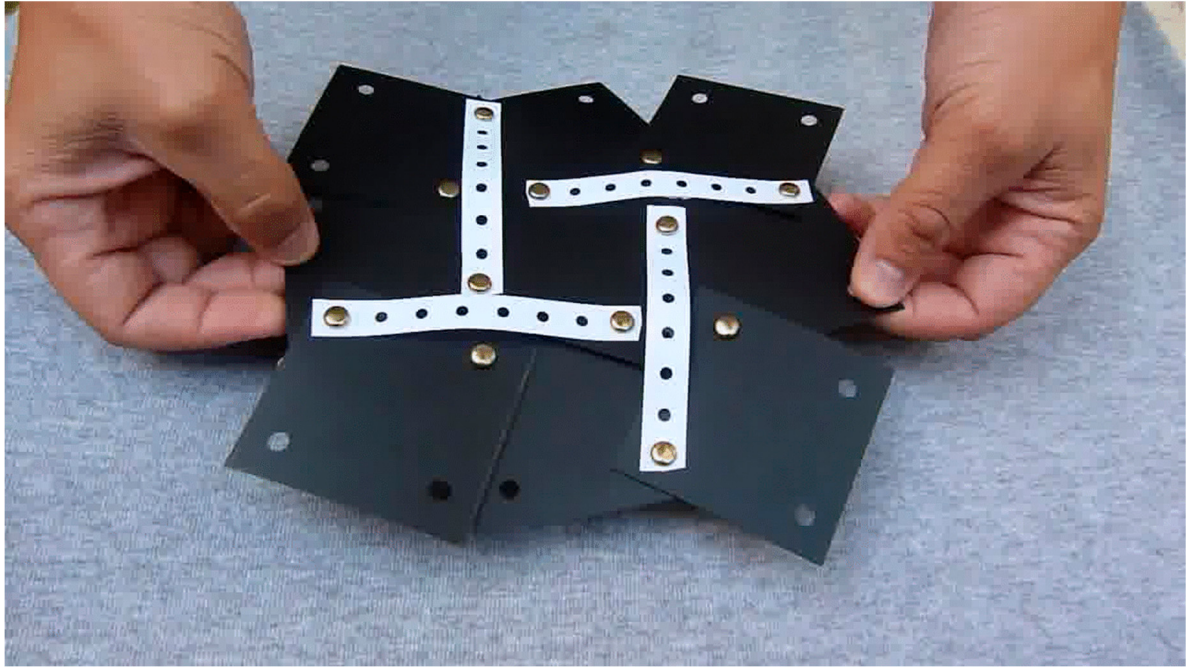

**Figure S1.** Animation of the Proposed Auxetic Mechanism.

Supplement: Supplementary file 1 [file materials-13-00079-s001.pdf]
